# Supplementary figures and images for: Microscopic vascular invasion may not be associated with survival of patients undergoing resection for solitary hepatoma of ≤ 2 cm
Source: PLoS One. 2023 Feb 9;18(2):e0281154. doi: 10.1371/journal.pone.0281154 (PMC9910699; doi:10.1371/journal.pone.0281154)

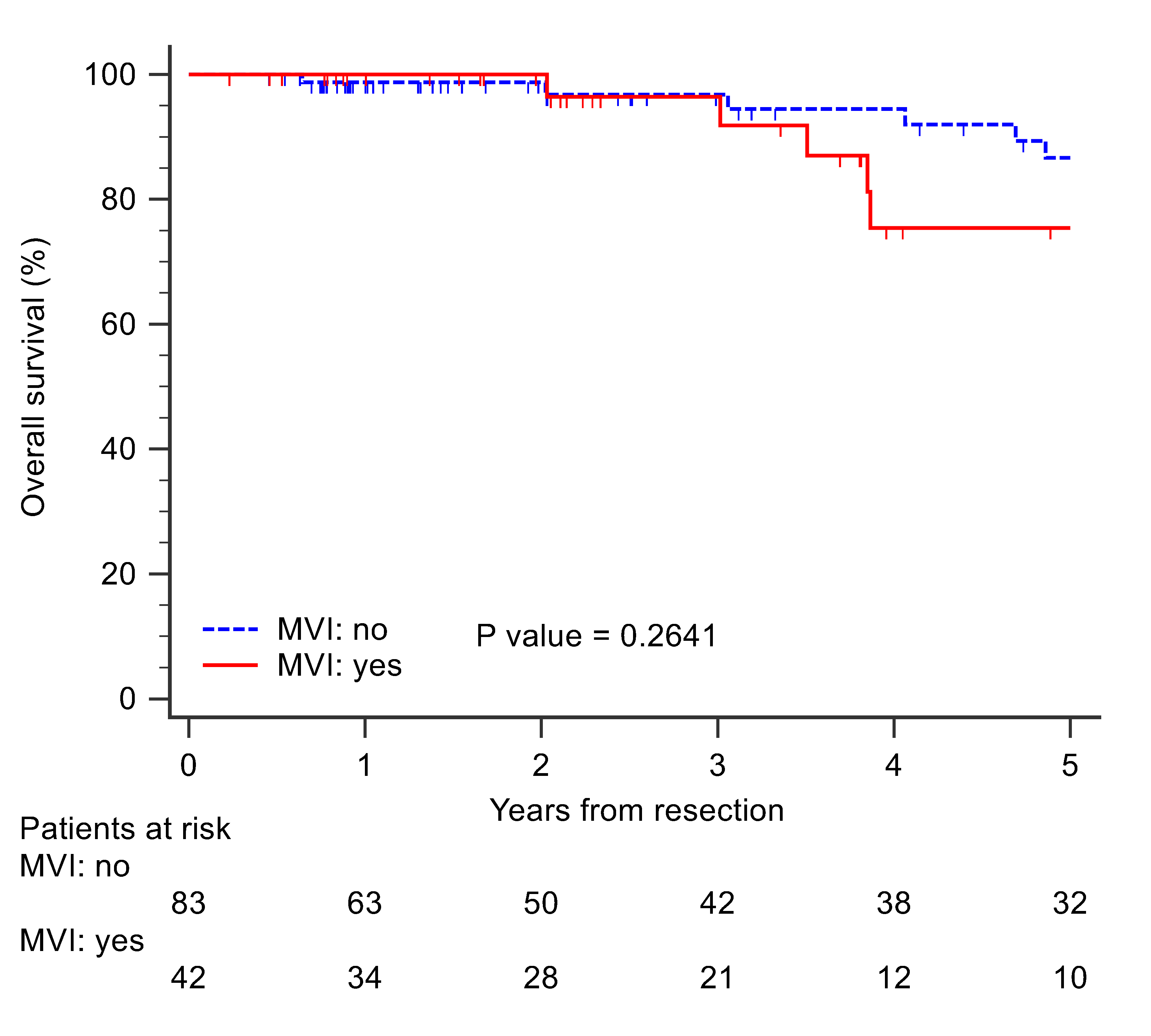

Supplement: S2 Fig — (TIF) [file pone.0281154.s002.tif]

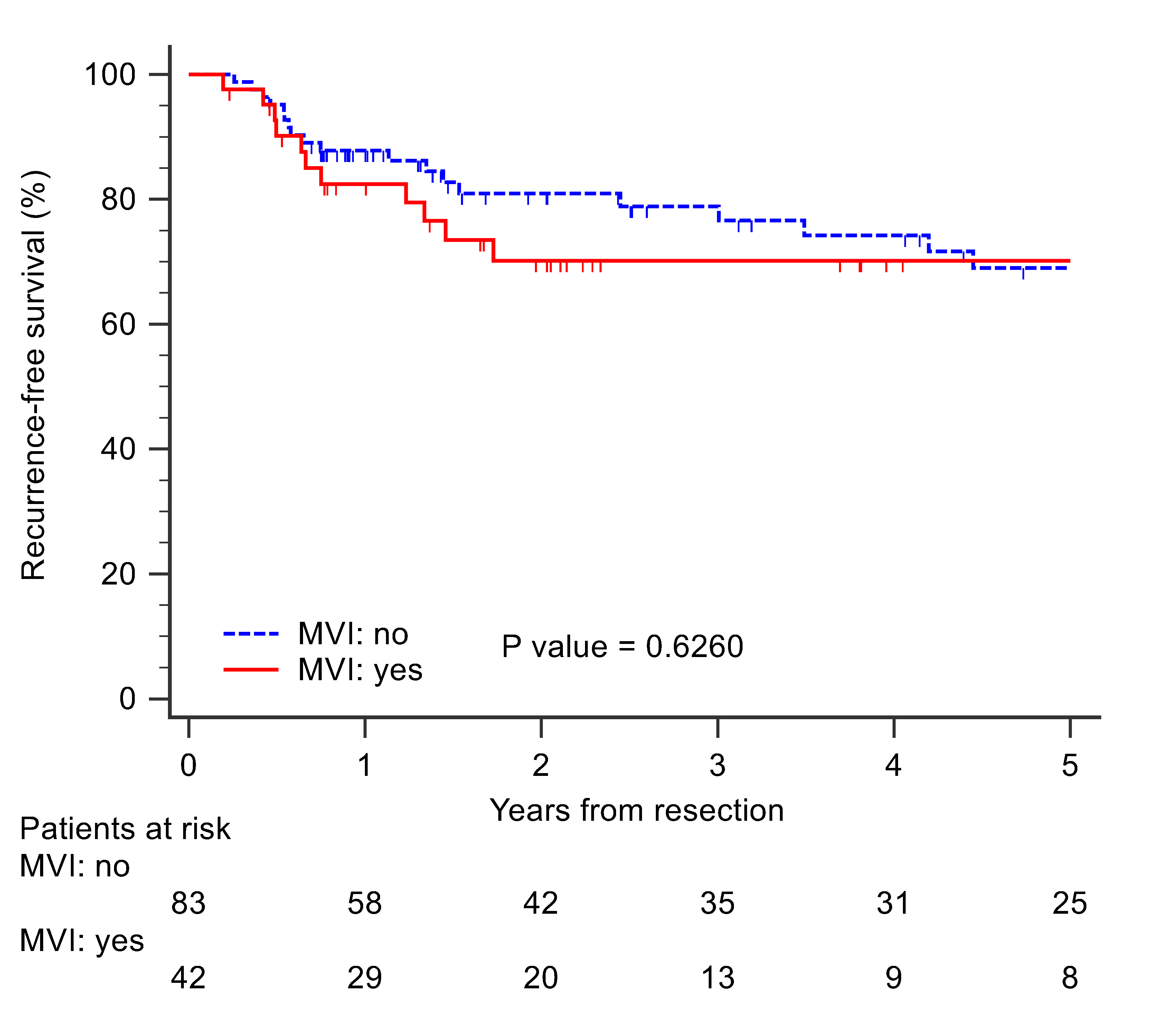

Supplement: S3 Fig — (TIF) [file pone.0281154.s003.tif]

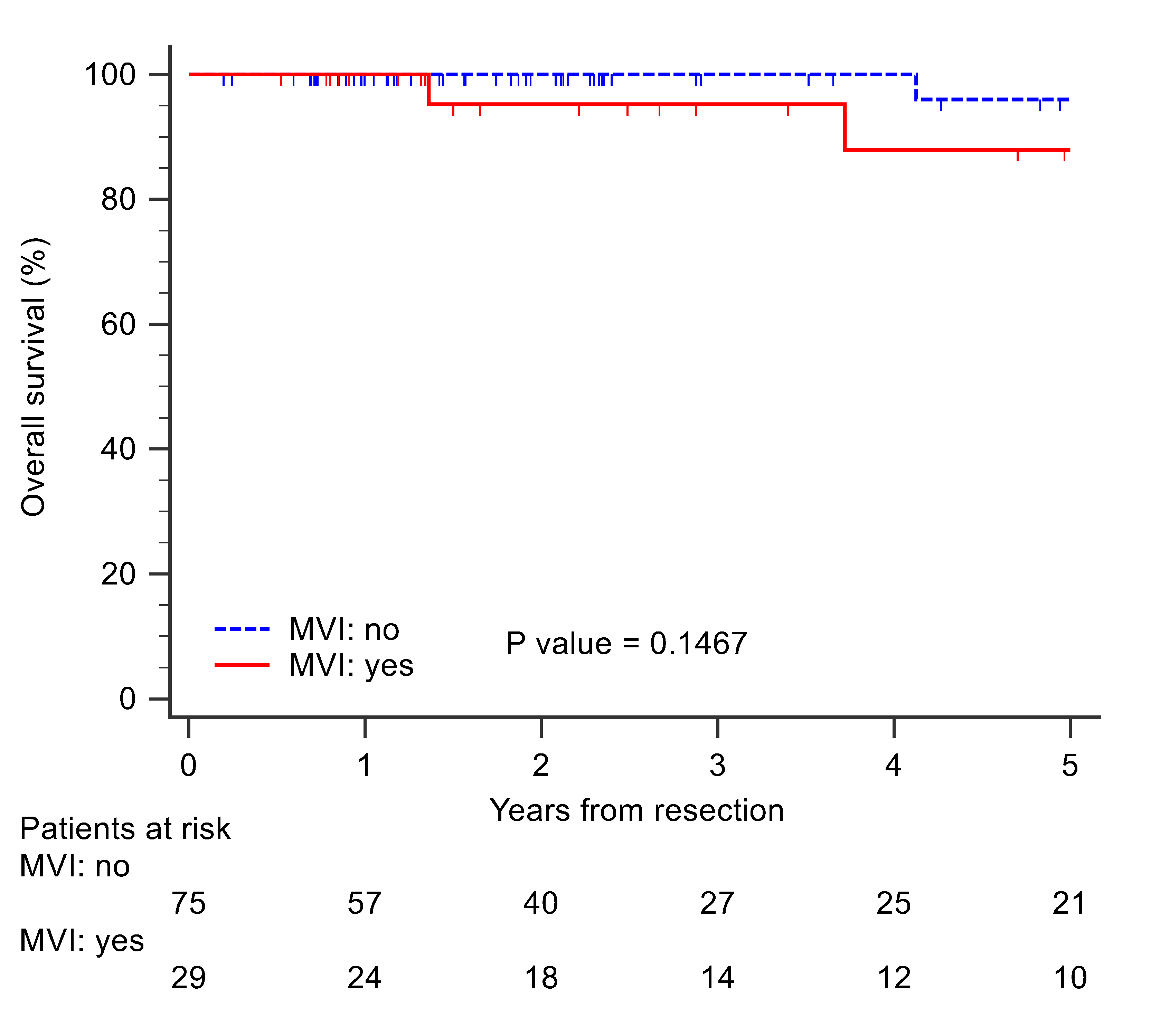

Supplement: S4 Fig — (TIF) [file pone.0281154.s004.tif]

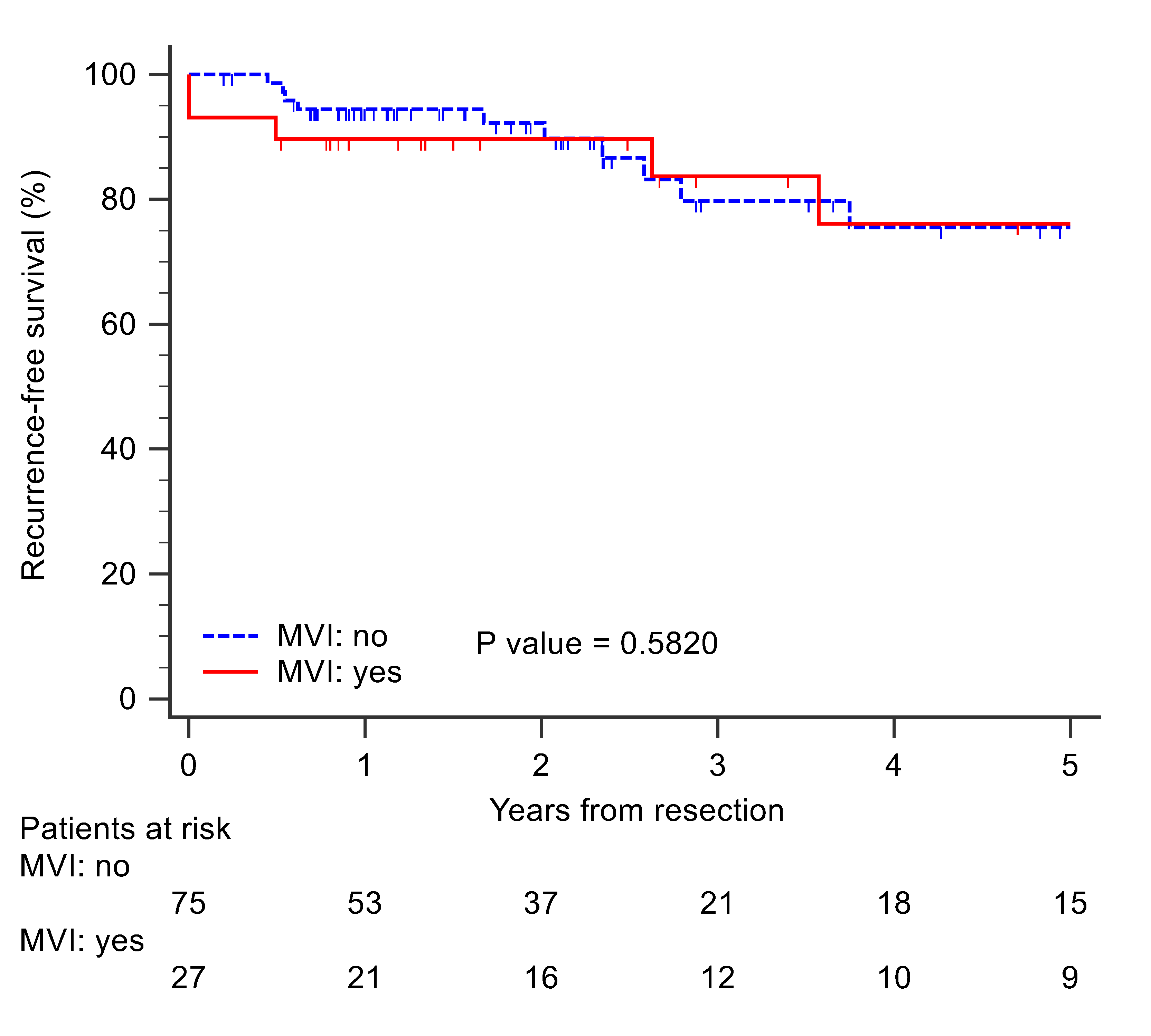

Supplement: S5 Fig — (TIF) [file pone.0281154.s005.tif]

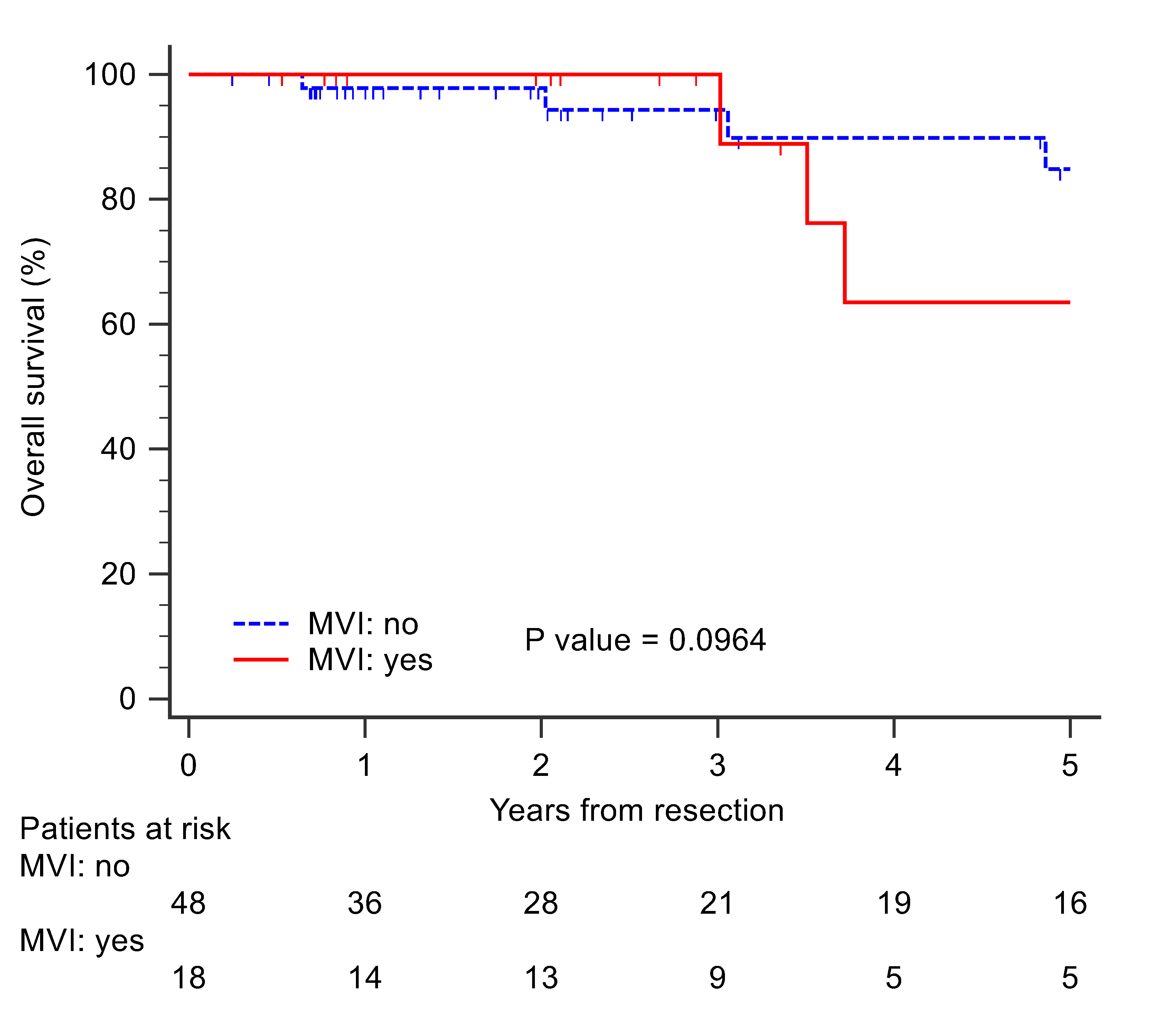

Supplement: S6 Fig — (TIF) [file pone.0281154.s006.tif]

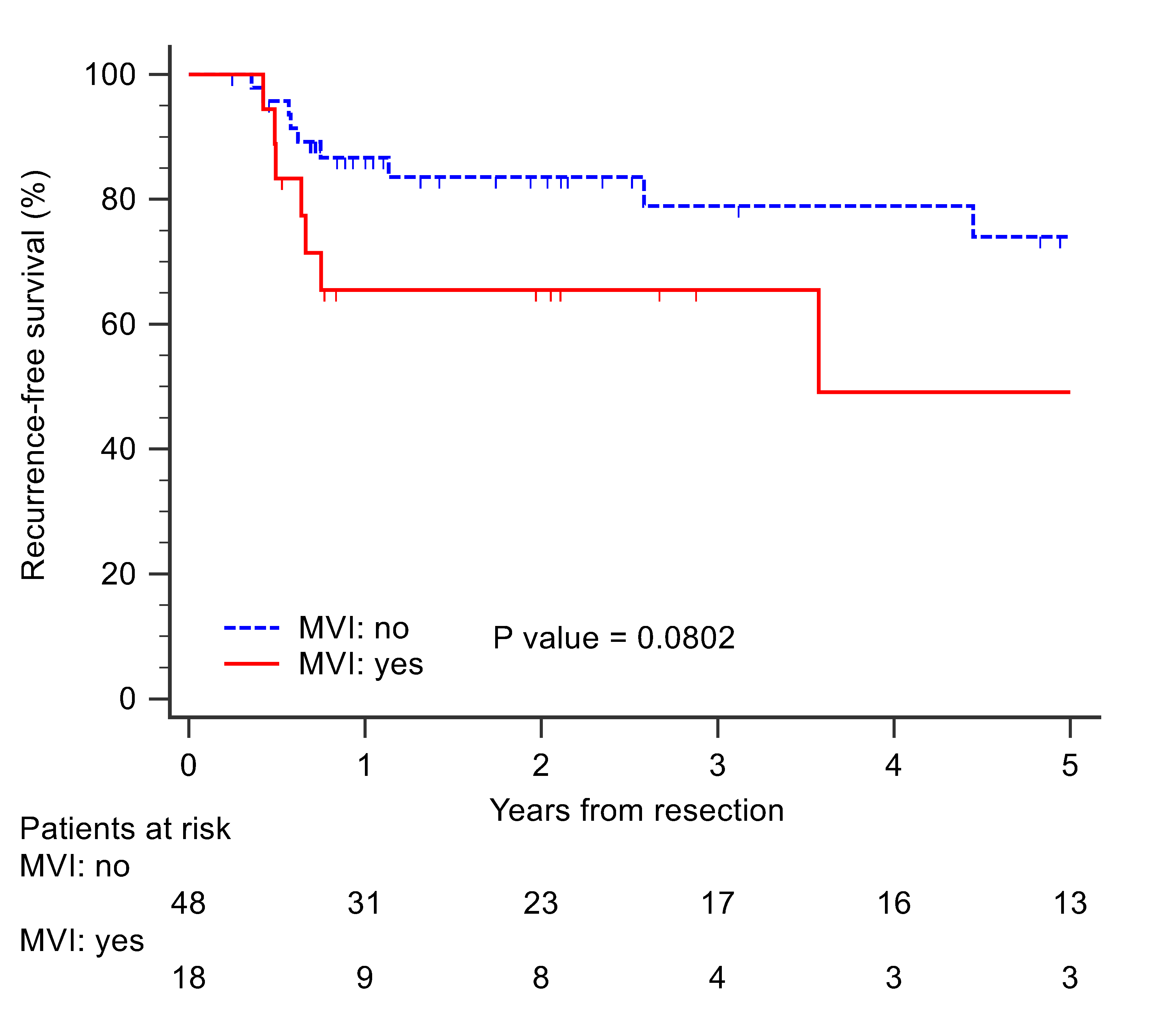

Supplement: S7 Fig — (TIF) [file pone.0281154.s007.tif]

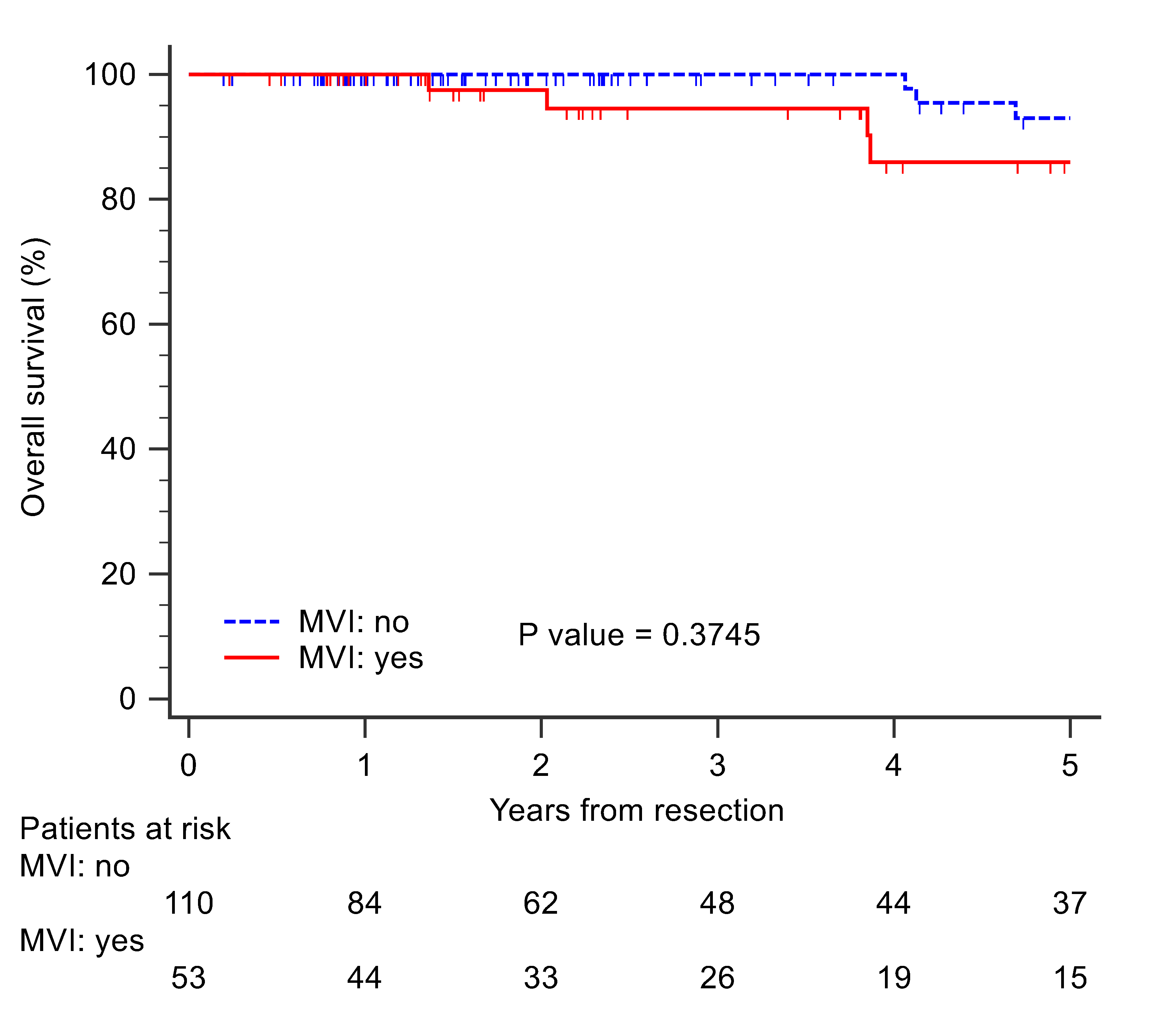

Supplement: S8 Fig — (TIF) [file pone.0281154.s008.tif]

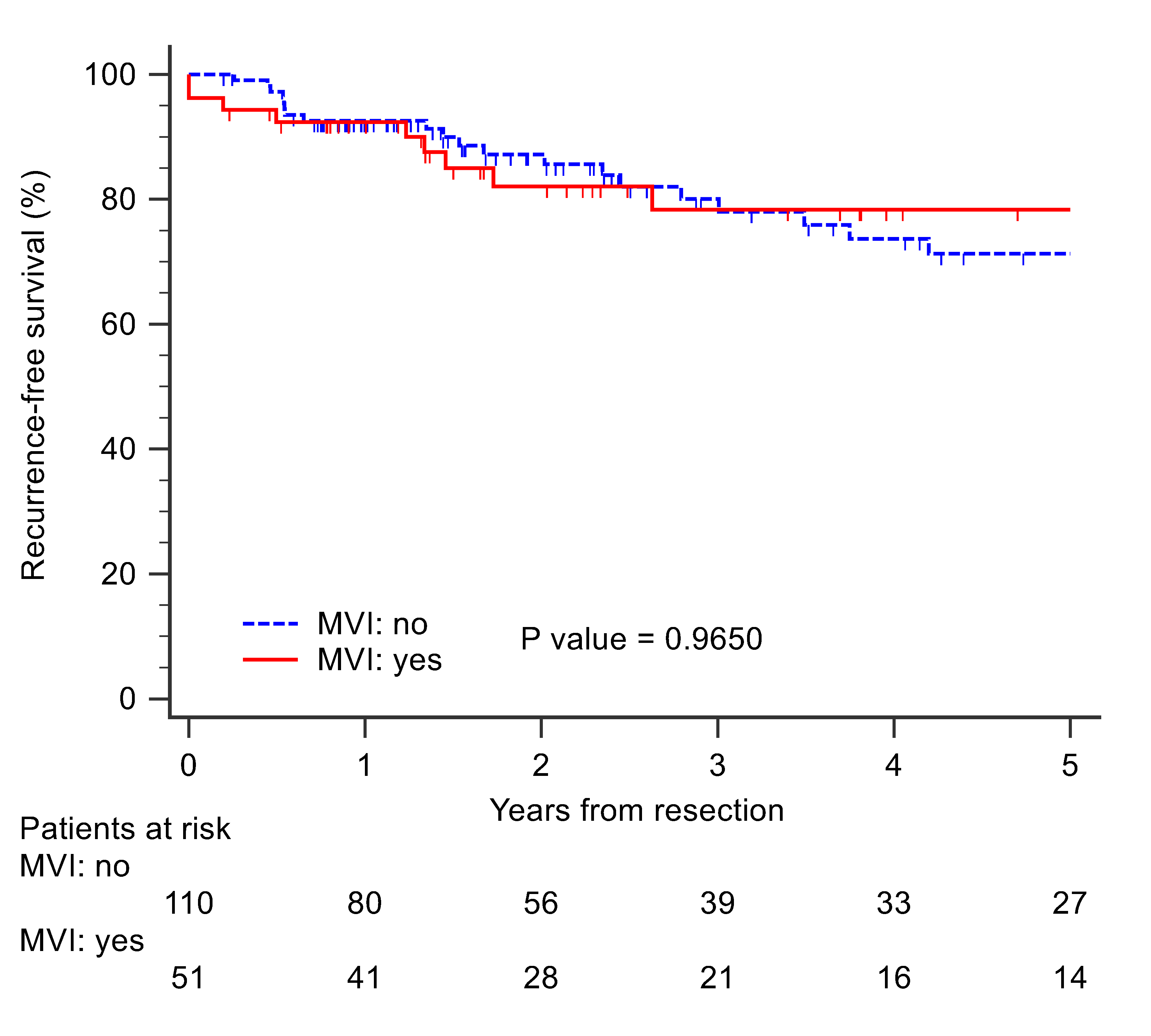

Supplement: S9 Fig — (TIF) [file pone.0281154.s009.tif]

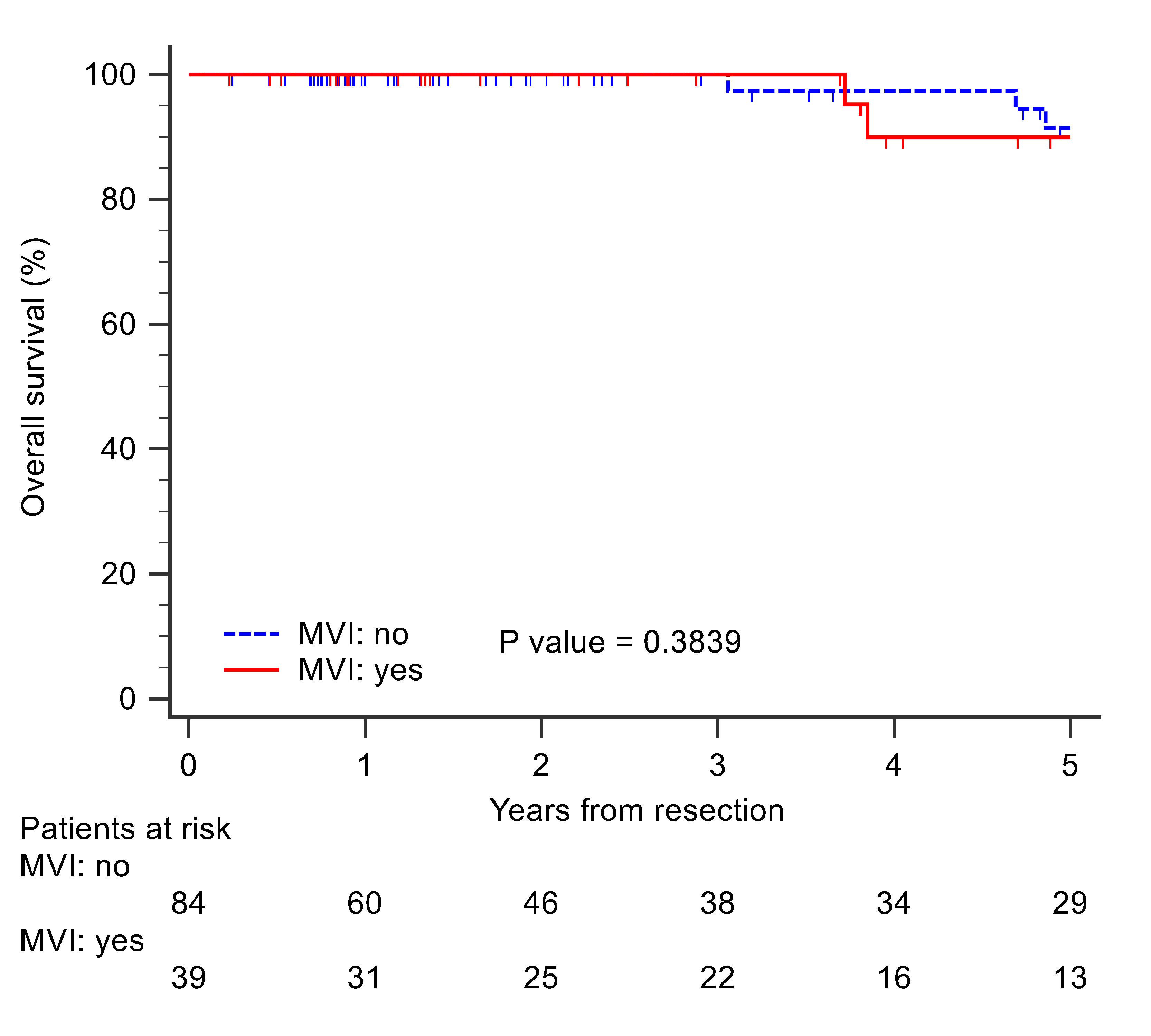

Supplement: S10 Fig — (TIF) [file pone.0281154.s010.tif]

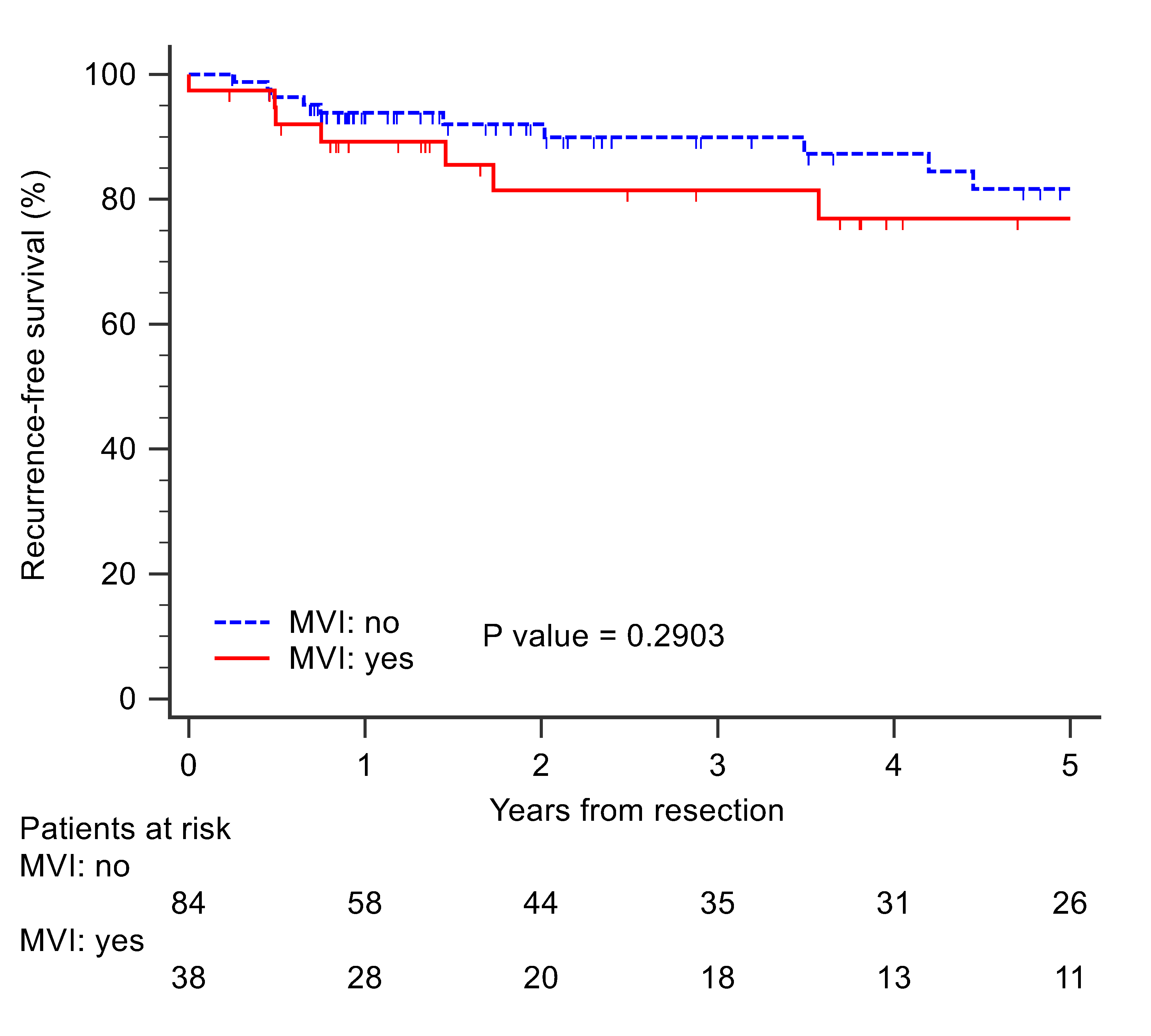

Supplement: S11 Fig — (TIF) [file pone.0281154.s011.tif]

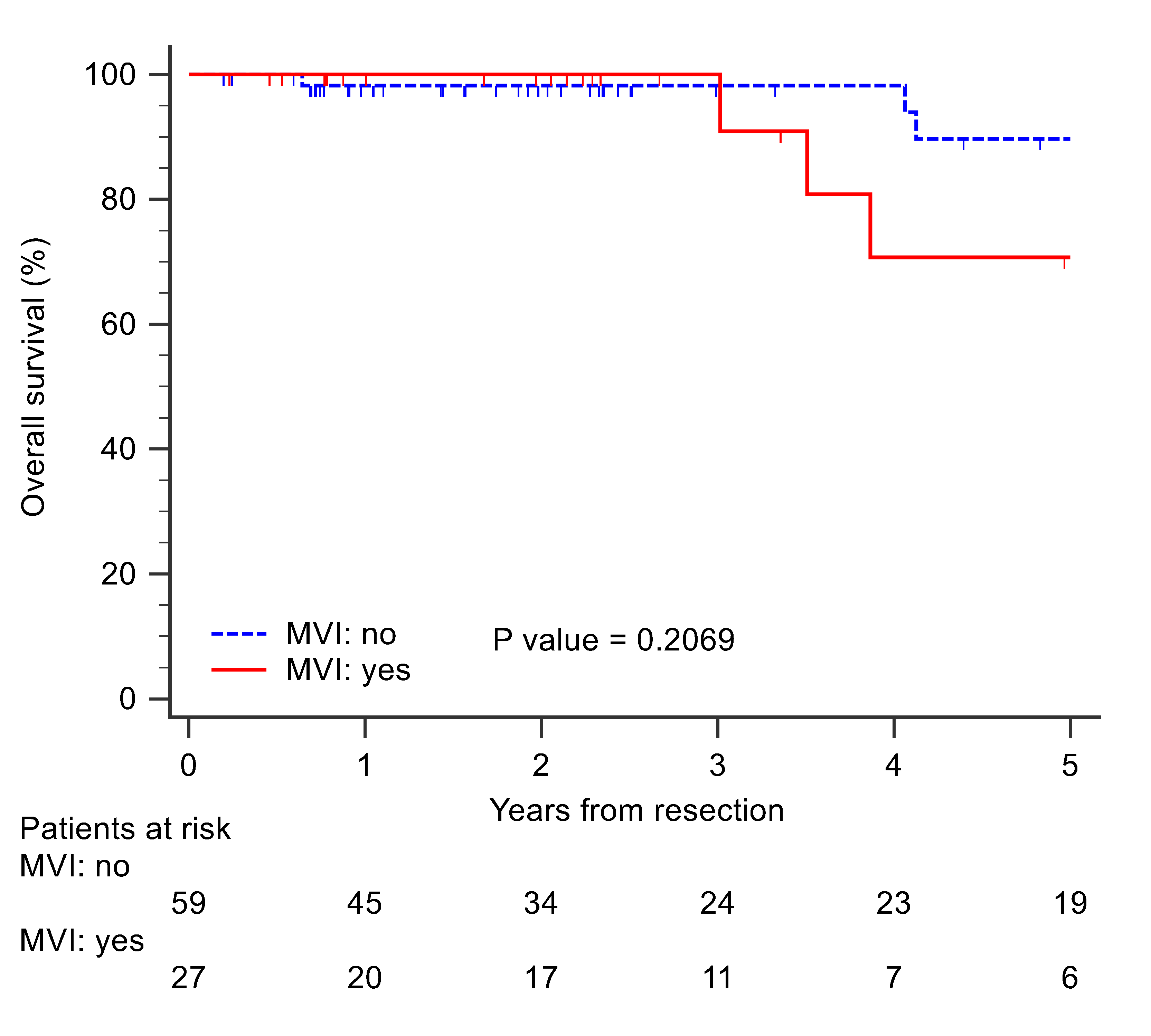

Supplement: S12 Fig — (TIF) [file pone.0281154.s012.tif]

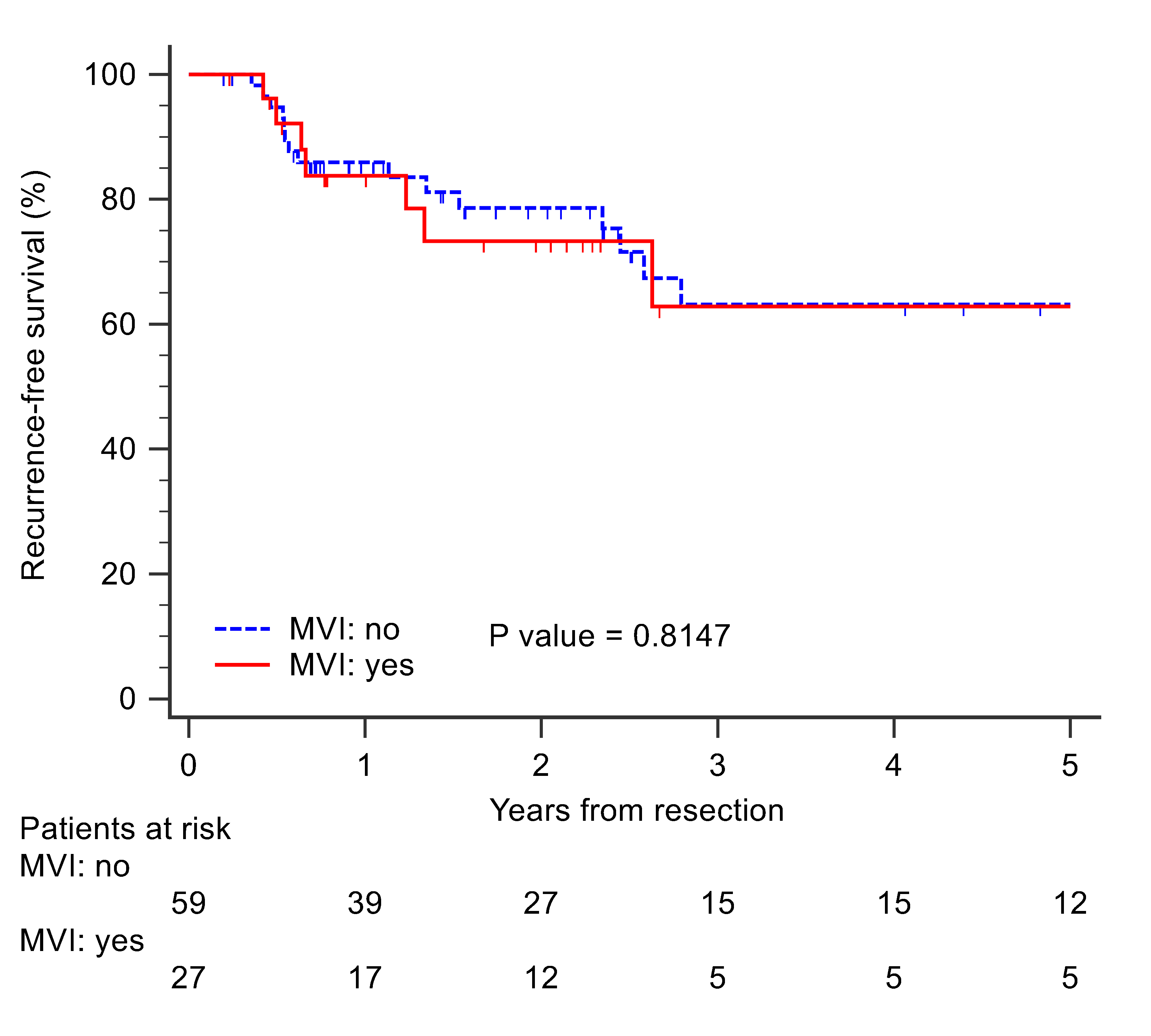

Supplement: S13 Fig — (TIF) [file pone.0281154.s013.tif]

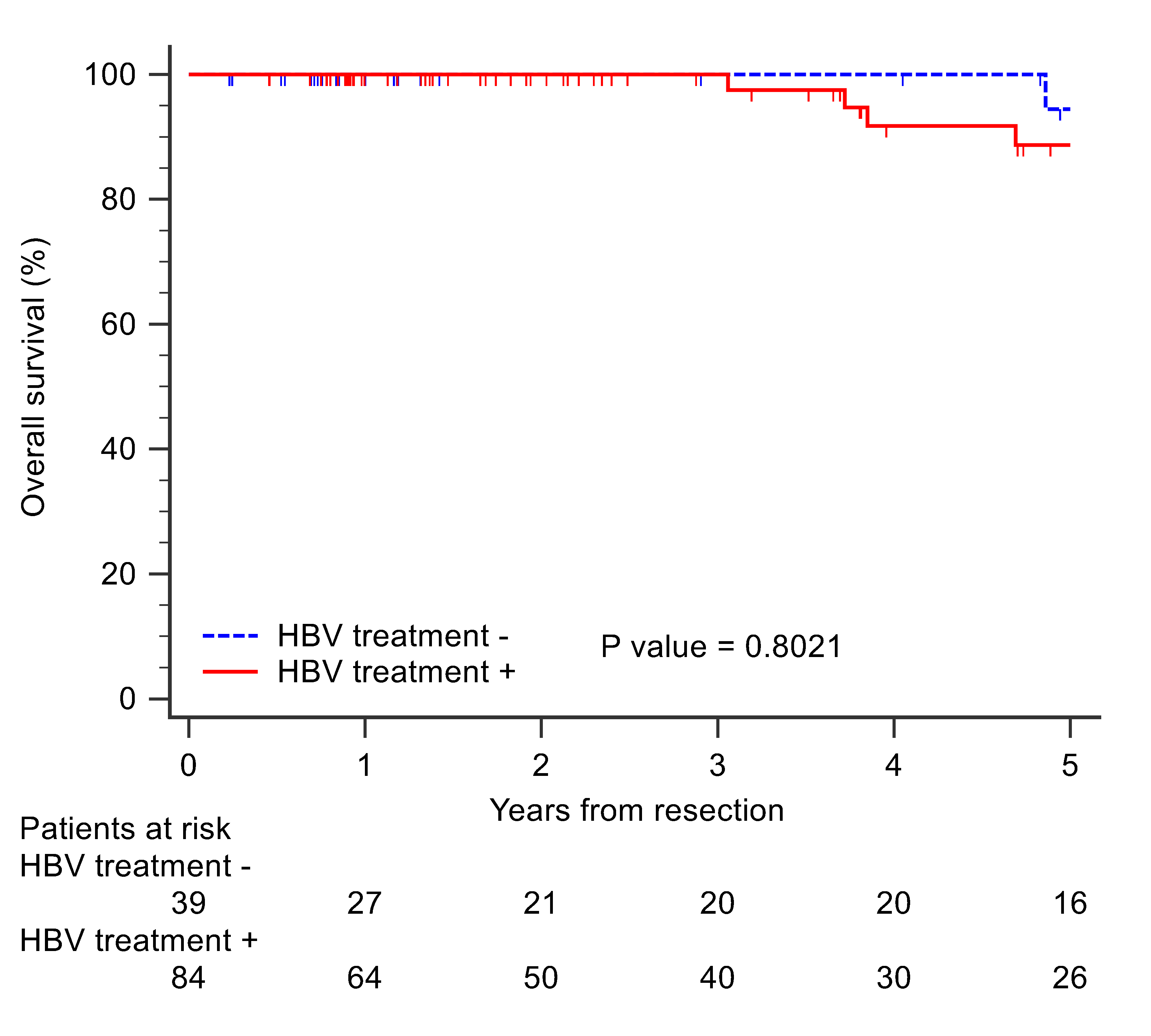

Supplement: S14 Fig — (TIF) [file pone.0281154.s014.tif]

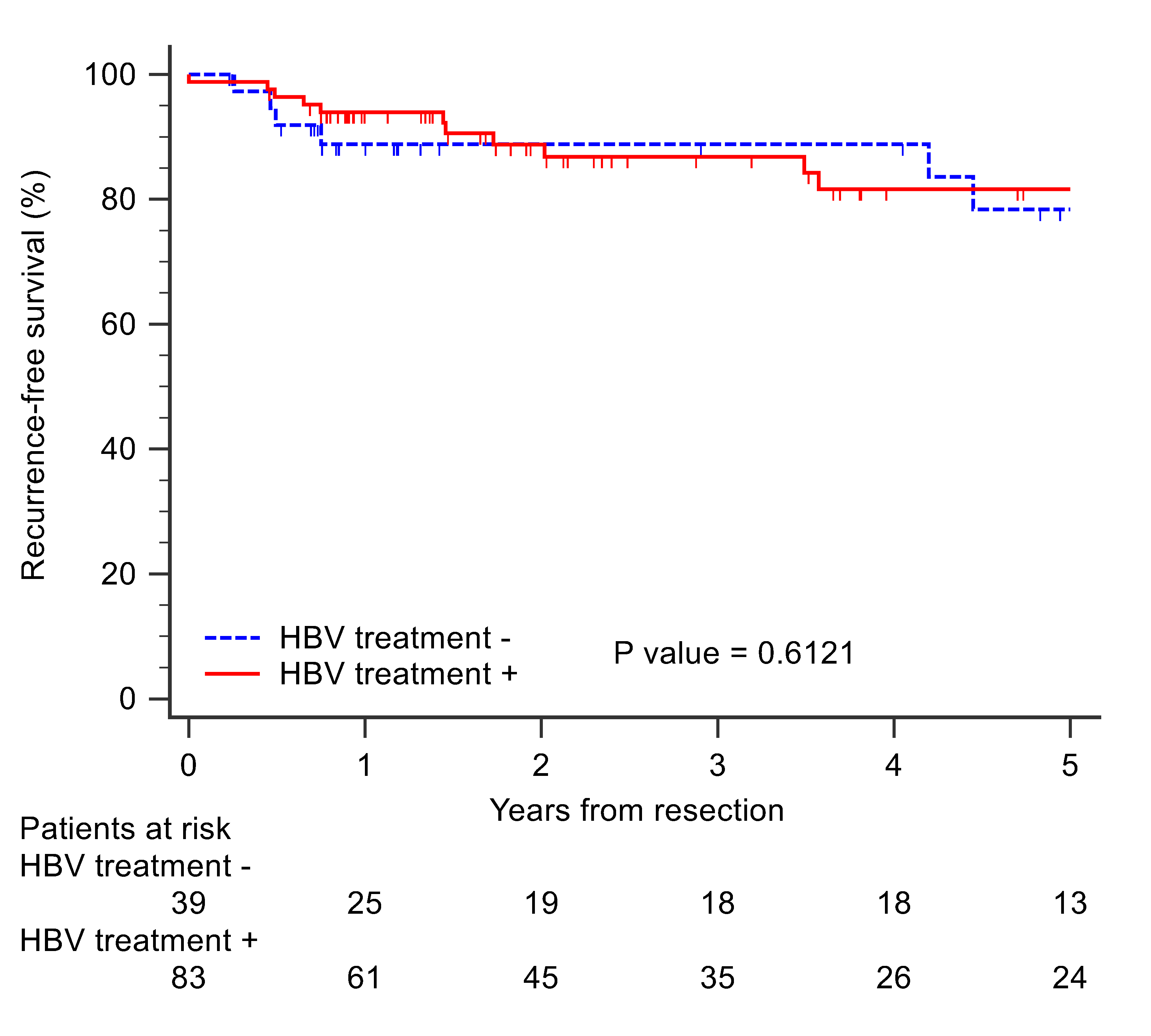

Supplement: S15 Fig — (TIF) [file pone.0281154.s015.tif]

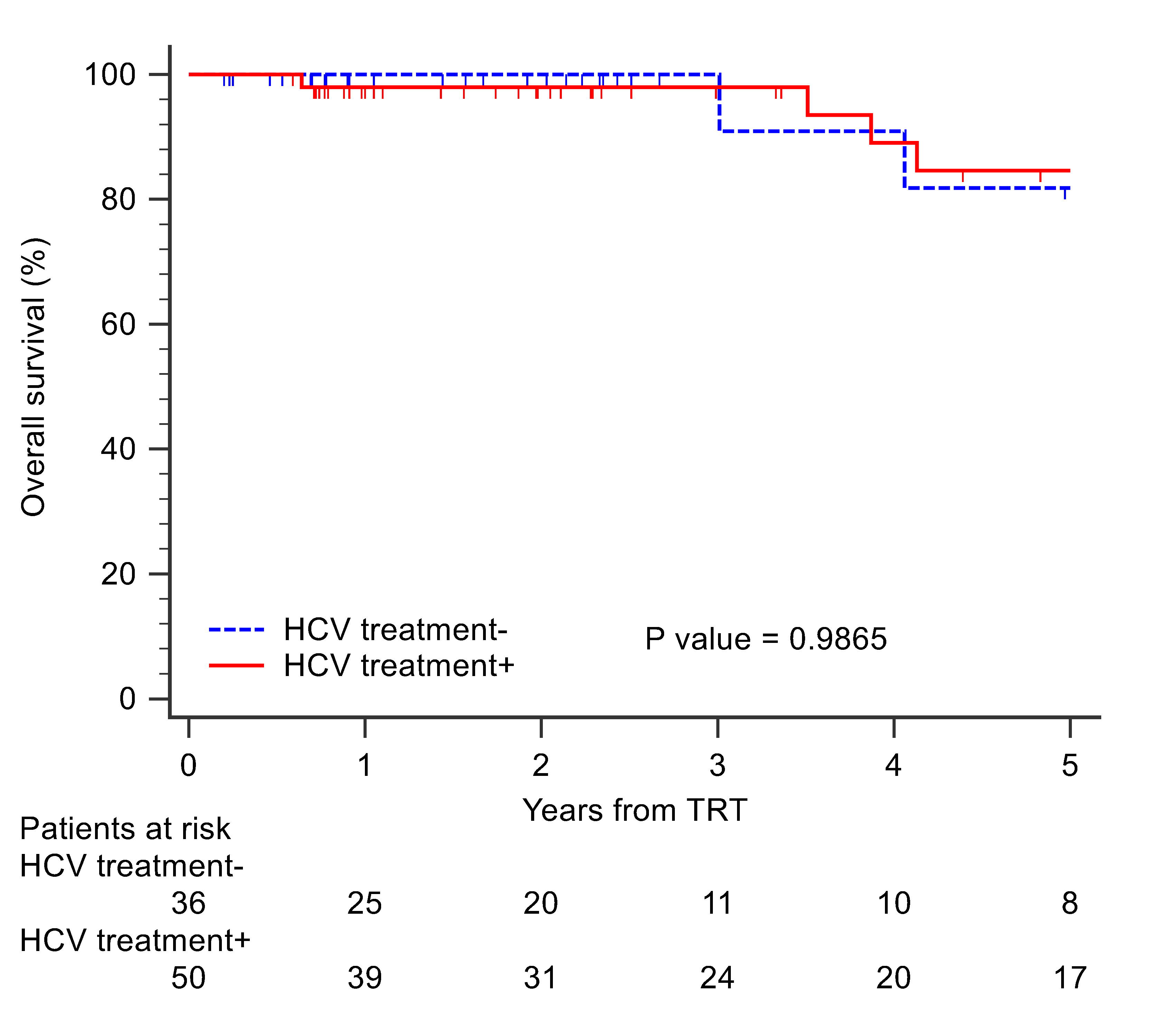

Supplement: S16 Fig — (TIF) [file pone.0281154.s016.tif]

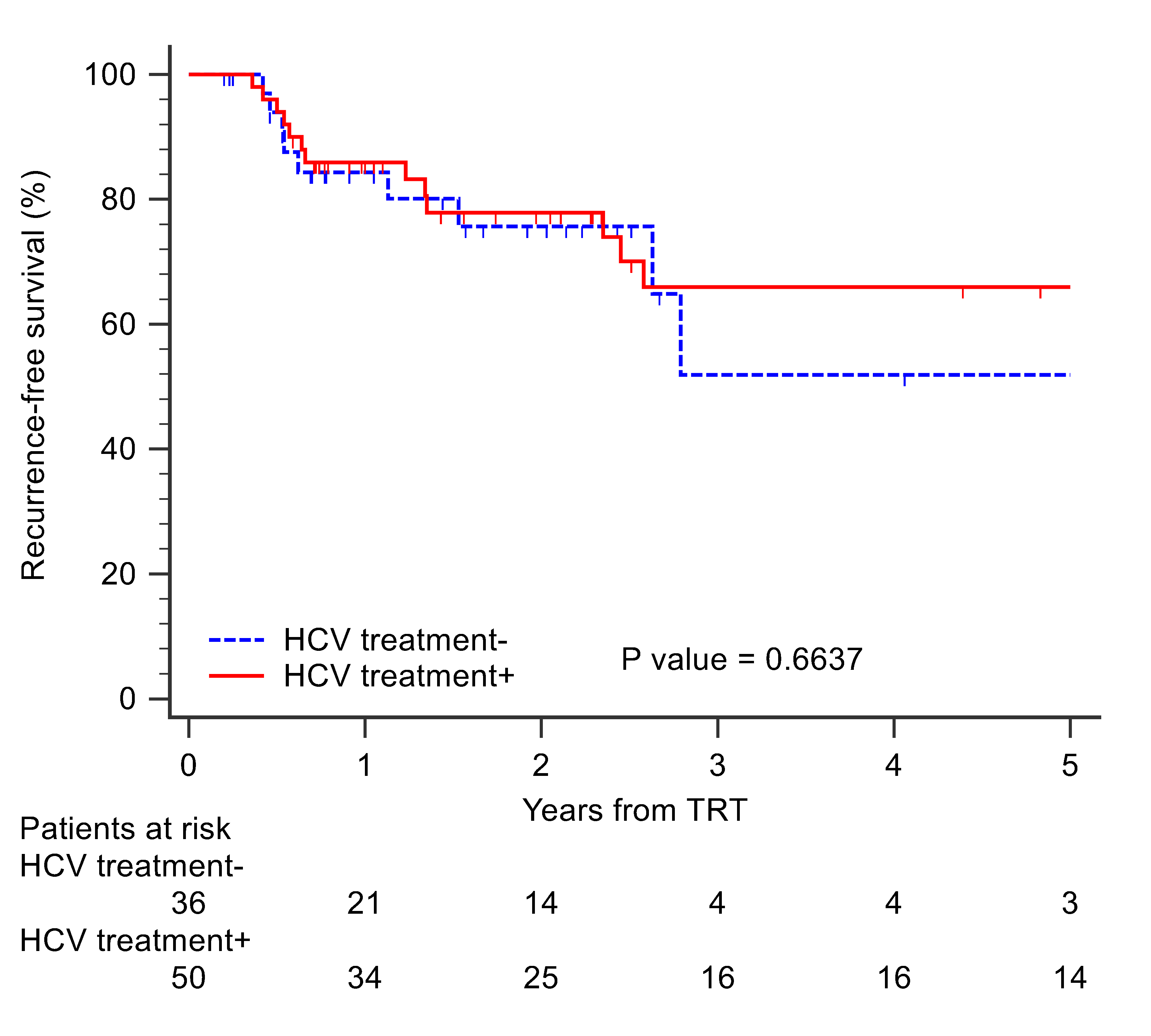

Supplement: S17 Fig — (TIF) [file pone.0281154.s017.tif]
